# Frog tongue acts as muscle-powered adhesive tape

**DOI:** 10.1098/rsos.150333

**Published:** 2015-09-30

**Authors:** Thomas Kleinteich, Stanislav N. Gorb

**Affiliations:** Zoological Institute: Functional Morphology and Biomechanics, Kiel University, Am Botanischen Garten 9, 24118 Kiel, Germany

**Keywords:** adhesion, biomaterials, feeding, amphibians, biomechanics

## Abstract

Frogs are well known to capture fast-moving prey by flicking their sticky tongues out of the mouth. This tongue projection behaviour happens extremely fast which makes frog tongues a biological high-speed adhesive system. The processes at the interface between tongue and prey, and thus the mechanism of adhesion, however, are completely unknown. Here, we captured the contact mechanics of frog tongues by filming tongue adhesion at 2000 frames per second through an illuminated glass. We found that the tongue rolls over the target during attachment. However, during the pulling phase, the tongue retractor muscle acts perpendicular to the target surface and thus prevents peeling during tongue retraction. When the tongue detaches, mucus fibrils form between the tongue and the target. Fibrils commonly occur in pressure-sensitive adhesives, and thus frog tongues might be a biological analogue to these engineered materials. The fibrils in frog tongues are related to the presence of microscopic papillae on the surface. Together with a layer of nanoscale fibres underneath the tongue epithelium, these surface papillae will make the tongue adaptable to asperities. For the first time, to the best of our knowledge, we are able to integrate anatomy and function to explain the processes during adhesion in frog tongues.

## 1. Introduction

The evolution of a muscular tongue has been a major innovation of terrestrial vertebrates [1,2] and is directly related to the emergence of a wide variety of feeding modes on land [3]. Especially, the projectile tongues of chameleons [4–9], several salamander species [10–15] and many species of frogs [16–21] have received considerable attention because of their ability to reach distant prey items at high velocities. Tongue adhesion generally is a highly dynamic process, and the time spent for contact formation between the tongue and the target is in the range of only a few milliseconds [7,11,17,22]. For frogs, the forces acting on the tongues during impact and retraction can even be beyond the body weight of the animals [22].

Despite the remarkable performances of projectile tongues in vertebrates as biological high-speed adhesive systems, little is known on the actual cause of their adhesive properties. In chameleons, it was demonstrated that a negative pressure can be generated inside the specialized tip of the tongue that forms a pouch [4]; in frogs and salamanders, the mechanism behind tongue adhesion is unknown. Based on the force over time relationship during detachment of the tongue of the horned frog *Ceratophrys* sp. from a glass slide, we hypothesized that frog tongues may act as pressure-sensitive adhesives (PSAs) [22], which is a class of materials comprising commonly used adhesives, such as sticky tape [23–25].

While we determined the forces acting during tongue adhesion in frogs in a previous study [22], here we went into detail and focused on the adhesive mechanism of a tongue against a smooth surface. We managed for the first time to actually capture the processes at the interface between a projected frog tongue and a target *in vivo*. The aim of this study is to explain the contact mechanical behaviour of adhesive projectile tongues in frogs by integrating *in vivo* observations of the processes during tongue adhesion with a detailed anatomical description at multiple organizational levels ranging from the tongue itself to nanoscale tongue elements.

## 2. Methods

### 2.1 Specimens and housing

Experiments to film the contact dynamics were performed with three adult specimens of horned frogs (Anura: Ceratophryidae: *Ceratophrys* sp.). One frog represented the species *Ceratophrys cranwelli*, two individuals belonged to the so-called fantasy colour morph, which does not occur in the wild and is achieved by crossing *C. cranwelli* with *Ceratophrys cornuta*. All specimens were captive bred individuals and purchased from the pet trade. These frogs are fossorial sit-and-wait predators native to Argentina, Bolivia, Brazil and Paraguay [26]. The animals were individually housed at temperatures of 26–29^°^C during the day and 24–26^°^C at night. Each terrarium was filled with loose substrate to a depth of approximately 5 cm in which the frogs could bury themselves. Usually, the frogs were found to be half buried and only occasionally, they moved to a different location. We moistened the terrariums daily to sustain a relative humidity of approximately 70–80%. Before the onset of the experiment, the animals were fed twice a week an alternating diet of crickets (*Gryllus bimaculatus*), grasshoppers (*Schistocerca gregaria*), wax-worms (*Zophobas morio*), earthworms (*Lumbricus terrestris*) and rodents (*Mus musculus*).

For the description of the tongue anatomy, two preserved adult specimens of *Ceratophrys ornata* were made available by the Zoological Museum Hamburg (ZMH A11916 and ZMH A11917).

### 2.2 Contact dynamics experiment

We filmed the tongue contact dynamics during the normal feeding routine of the frogs. During each experimental session, a maximum of four trials per individual was recorded. Overall, we filmed 25 tongue contacts.

**Figure 1.**
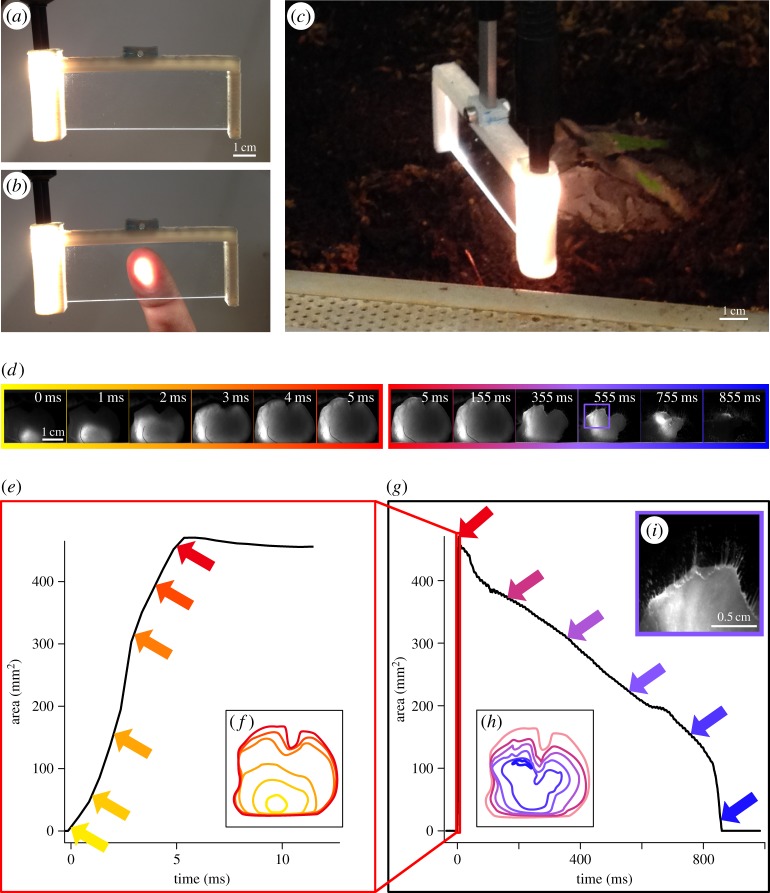
Contact dynamics of frog tongues. We used the effect of frustrated total internal reflection to visualize frog tongues during contact with a glass slide. For this, we created a frame that fitted a glass slide and that allowed light from a gooseneck light source to be guided into the glass. Light was totally reflected within the glass (*a*) and was only transmitted at contact regions (*b*; here demonstrated with a fingerprint). We then mounted these frames in the terrariums with the frogs (Ceratophryidae: *Ceratophrys* sp.) (*c*) and presented a cricket to the frogs behind the glass slide. If the frogs tried to catch the cricket, their tongues adhered to the glass and the illumination of the glass slide at the contact area was captured with a high-speed video camera at 2000 frames per second (*d*). (*e*,*g*) The development of the tongue contact area over time for contact formation (*e*) and release (*g*) for the exemplary experimental trial shown in (*d*); arrowheads correspond to individual frames in (*d*). Tongue contact formation happens at least one order of magnitude faster than detachment. (*f*,*h*) Tongue shape changes during contact; for this, we traced the outlines of the tongue for selected frames in the high-speed video recordings. Different colours for the outlines relate to different frames and correspond to the coloured arrowheads in (*e*,*g*). Contact formation progresses from proximal regions of the tongue distally (*f*); contact release happens from the outside of the tongue contact area to the inside (*h*). During detachment, mucus fibrils are clearly visible (*i*).

For visualization of tongue contact formation and release, we created a frame that fitted a regular glass object slide by using the three-dimensional design software SketchUp Make v. 14.1 (Trimble Navigation Limited, available at www.sketchup.com). This frame was open to one side to easily change the glass slides after each experimental trial. The other side of the frame was designed as a cylinder that had the same diameter as the gooseneck of a cold light source (Zeiss CL 1500 Eco, Carl Zeiss AG, Germany). With the light source attached, light was directed into the glass slide (figure 1*a*). Further, a mount for a force transducer (World Precision Instruments, Sarasota, FL, USA) was modelled onto the frame, but owing to the rigidity of the gooseneck, simultaneous force measurements could not be performed during contact area filming, and this mount was only used to fixate the frame during the experiment. We created real frames by rapid prototyping the three-dimensional designed model with ABS plastic on a CubeX Duo three-dimensional printer (3D Systems, Rock Hill, SC, USA).

For experimental trials, the frame was placed into the terrariums of the frogs in a vertical position approximately 2 cm in front of the animals (figure 1*c*). Generally, the frogs did not move and remained half buried during the set-up procedure of the experiment. We then placed a cricket immediately behind the glass slide and held it in place with tweezers. As the frogs tried to capture the cricket, their tongues attached to the glass slides (figure 1*d*). Owing to the effect of frustrated total internal reflection, light was transmitted from the illuminated glass slides only at regions where the tongues actually were in contact with the glass. We filmed this light transmission at the tongue contact interface with a Photron Fastcam SA1.1 high-speed-video camera (Photron Europe Limited, Bucks, UK) at 2000 frames per second. To avoid a negative learning effect for the frogs, we fed them the crickets after each experimental trial.

We filmed the tongue contact at an angle of approximately 20–45^°^ relative to the axis perpendicular to the glass slide to avoid having the contact area of the tongue obscured by the presence of the cricket on the side of the glass slide facing the high-speed video camera. This resulted in a slight perspective distortion of the glass surface in the recorded videos and in a gradual decrease of brightness of the illuminated contact area from the side facing the camera to the far side. We corrected for the distortion by using the *Landmark Correspondence* plugin [27] of the FIJI distribution of the open source image analysis software ImageJ (available at www.fiji.sc). As reference shape for distortion correction, we used a rectangle that had the exact height/width ratio as the glass slides. After distortion correction, we defined regions of interest in the datasets to exclude structures that were visible in the high-speed video recordings but that were not in contact with the glass surface (e.g. the frog moving behind the glass slide or the illuminated ABS plastic frames). Within these regions of interest, we used the *Analyse Particles* function of ImageJ to automatically measure the size of the contact area for each recorded frame.

Statistical analysis of the resulting data for contact area over time was performed with the open source statistical computing package R v. 3.1.1 (available at www.r-project.org). For each experimental trial, we plotted the development of the contact area over time. We extracted the following variables: (i) maximum contact area, (ii) duration of contact formation; i.e. the time from the first contact to the time at which the maximum contact area was measured, and (iii) duration of detachment; i.e. the timeframe from maximum contact to the final release of the tongue from the glass slide. From visual inspection of the area over time relationships, it appeared that for substantial amounts of time the decrease of contact area over time is almost perfectly linear (figure 1*g*). We visually defined the onset and end of this linear phase and calculated the relative portion of the duration of contact breakage in which this linear phase was observed. Further, we fitted a linear regression line of contact area over time to this particular phase of contact release. The slope of this regression line is a measure on how fast the contact breaks.

### 2.3 Microcomputed tomography imaging

We used a multi-scale approach for microcomputed tomography (micro-CT) imaging: (i) we micro-CT scanned entire frogs, (ii) we micro-CT scanned isolated tongues which we dissected from the specimens, and (iii) we prepared small pieces of tongue tissue that we scanned separately at highest resolution. To visualize soft tissues with micro-CT imaging, we used 4% LUGOL’s iodine and potassium iodine solution as the staining agent following the protocol by Metscher [28], but we increased the staining time to two weeks to stain entire frogs. All micro-CT scans were performed in distilled water. For this, frog specimens were placed in plastic containers, tongues were mounted inside Falcon^®^ tubes, and pieces of tongue surface tissue were squeezed into the tips of water-filled pipettes and wrapped with laboratory film (Parafilm M^®^, Bemis Company Inc., Oshkosh, WI, USA).

For micro-CT scanning, we used a Skyscan 1172 desktop micro-CT scanner (Bruker micro-CT, Kontich, Belgium). The frog specimens were scanned at a source voltage of 100 kV and a source current of 100 μA; isolated tongue specimens were scanned with 70 kV at 140 μA; small pieces of tongue surface tissue were scanned at 40 kV and 250 μA. Micro-CT scanning resulted in image stacks of X-ray projections; between each projection, the specimen was rotated by 0.3^°^, respectively 0.25^°^ for high-resolution scans of tongue pieces. From these X-ray projections, stacks of virtual cross sections through the specimens were reconstructed with the software NRecon (Bruker micro-CT, Kontich, Belgium). The reconstructed cross-sectional images had pixel resolutions of 26.7 μm for entire animal scans, 22.1 μm for tongue scans and 0.9 μm for scans of tongue tissue pieces. The reconstructed micro-CT datasets for each scan are available online at http://dx.doi.org/10.5061/dryad.066mr. The stacks of cross-sectional images were imported as volumetric data into the three-dimensional visualization software package Amira v. 5.4.2 (FEI) for three-dimensional rendering.

### 2.4 Scanning electron microscopy

After micro-CT imaging, we prepared pieces from the isolated frog tongues for scanning electron microscopy. For this, we first dehydrated the specimens by a stepwise transfer to 100% ethanol (with steps of 30%, 50%, 70%, 90%; each step was maintained for 24 h). During this process, the iodine potassium iodide stain was washed out from the specimens. After dehydration, the tongue specimens were critical point dried with a Quorum E3000 critical point drying system (Lewes, UK). In a first step, the specimen chamber was cooled down to 5^°^C, and the ethanol was substituted by liquid CO_2_. The specimen chamber was then heated to 40^°^C, which corresponded to a pressure of 95 bar and is beyond the critical point of CO_2_ (35^°^C, 80 bar). The pressure was then slowly released, whereas the temperature was kept constant at around 40^°^C, causing the CO_2_ to evaporate. After drying, we fractured one tongue fragment with a sharp razor blade. Prior to scanning electron microscopy, we coated the specimens with a 10 nm gold–palladium layer by using a Leica SCD05 Sputter Coater. For scanning electron microscopy, we used a Hitachi S-4800 scanning electron microscope at an accelerating voltage of 3 kV.

## 3. Results

### 3.1 Tongue contact dynamics

To visualize the processes at the interface between a frog tongue (*Ceratophrys* sp.) and a target, we applied the principle of frustrated total internal reflection by which light is transmitted out of an illuminated glass slide only at regions of contact with the tongue (figure 1*a*,*b*). This light transmission was then captured with a high-speed video camera at 2000 frames per second (figure 1*d*; electronic supplementary material, videos S1 and S2). Tongue impact happens rapidly, on average it takes only 19.2±16.1 ms (*n*=24) from the first contact to reach a peak contact area which is on average 331.8±151.7 mm^2^. The leading edge of contact formation always progresses from the proximal parts of the tongue, i.e. close to the connection with the tip of the lower jaw, distally towards the terminal tongue lobes (figure 1*d*,*f*).

Detachment is two orders of magnitude slower than contact formation and takes on average 1177.3±952.3 ms (*n*=24). During detachment, the tongue surface area shrinks from the outside towards the centre of the tongue (figure 1*h* and electronic supplementary material, video S2). We found that detachment starts with a sudden decrease of the tongue contact area that is then followed by a phase in which the contact area shrinks almost constantly over time (figure 1*g*). During this phase of detachment, fibrils are visible that maintain contact with the glass surface in regions where the main body of the tongue is already detached (figure 1*i* and electronic supplementary material, video S2). The phase of linear decrease of tongue surface area over time lasts then for on average 59.7±14.9% (*n*=24) of the total duration of the detachment phase. In all experimental trials herein, the relationship of contact area over time can well be represented by a regression line (adjusted *r*^2^>0.91 except for one trial with *r*^2^=0.69; *p*<2×10^−16^; electronic supplementary material, table S1). During the period of linear decrease, the attached tongue surface shrinks on average by 2.59±1.38 cm^2^ s^−1^ (*n*=25).

### 3.2 Morphology

**Figure 2.**
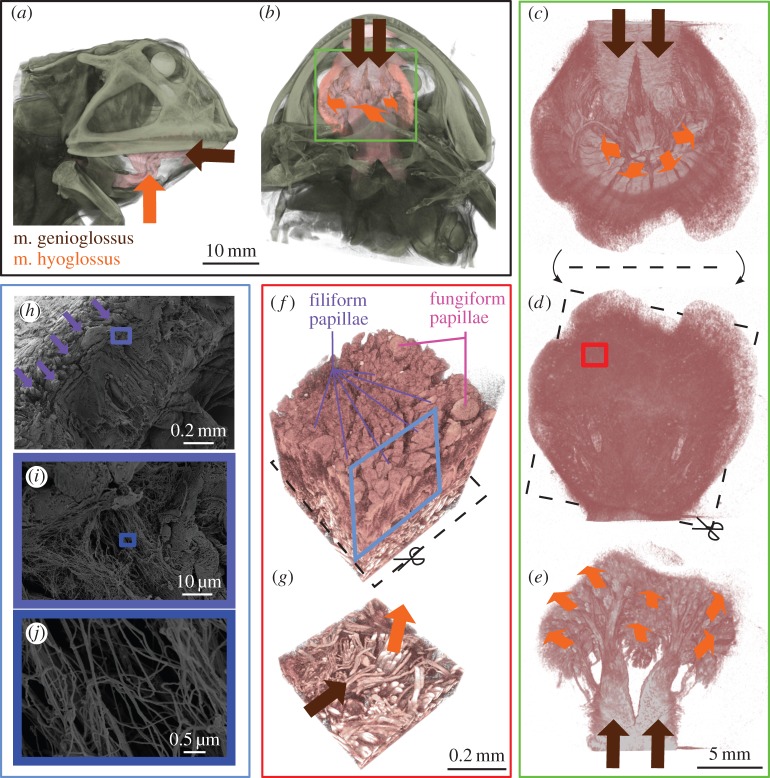
Tongue anatomy in *Ceratophrys ornata*. (*a*–*g*) high-resolution micro-computed tomography of frog tongues at the level of the organism (*a*, lateral view; *b*, ventral view), the organ (*c*, ventral view; *d*,*e*, dorsal views) and the surface (*f*,*g*, dorsal views). The body of the tongue is composed by two muscles, the m. genioglossus (brown arrowheads) and the m. hyoglossus (orange arrowheads); the orientation of the arrowheads depicts the fibre orientation of the muscles. The m. genioglossus is oriented parallel to the tongue surface, the m. hyoglossus inserts perpendicular to the tongue epithelium and the m. genioglossus. Both muscles show a branching pattern from proximal to distal and the fibres of the two muscles are tightly interwoven (*e*,*g*). The tongue surface is covered by two types of papillae: the larger and spherical fungiform papillae are surrounded by smaller and more hair-like filiform papillae. (*h*–*j*) Scanning electron microscopy of critical point dried and fractured pieces of frog tongue. Most of the filiform papillae are covered in mucus but some could clearly be identified along the fractured edge (*h*). Underneath the papillae, we found a network of thin fibres that were oriented in a perpendicular direction towards the tongue surface (*i*–*j*).

The tongue in *C. ornata* is built from two muscles: (i) the tongue-protracting musculus genioglossus (m. genioglossus) and (ii) the tongue-retracting musculus hyoglossus (m. hyoglossus). In *C. ornata*, fibres of the m. genioglossus originate from the lingual face of the rostral parts of the lower jaw and enter the tongue immediately dorsocaudad to the jaw symphysis (figure 2*b*). The m. genioglossus is oriented parallel to the tongue surface, and bundles of muscle fibres are branched from this muscle as it runs from proximal-to-distal parts of the tongue (figure 2*e*). The m. hyoglossus enters the tongue at its centre in the shape of a thick bundle of muscle fibres from which subsequently smaller subunits of muscle fibres are branched off to run towards the periphery of the tongue (figure 2*c*). The fibres of the m. hyoglossus run perpendicular to the tongue surface and thus perpendicular to the fibres of the m. genioglossus with which they are interwoven (figure 2). The dentrite-like pattern of the m. hyoglossus results in an even distribution of m. hyoglossus fibres over the tongue surface (figure 2*e*).

The dorsal tongue surface in *C. ornata* is covered by two different kinds of papillae (figure 2*f*) that are referred to as fungiform and filiform. Scanning electron microscopy shows that wide parts of the filiform papillae are covered by mucus (figure 2*h*). By fracturing critical point dried *C. ornata* tongue pieces, we found approximately 30 nm thin fibres that are mostly oriented perpendicular to the tongue surface underneath the dorsal tongue epithelium (figure 2*h*–*j*).

## 4. Discussion

For the first time, to the best of our knowledge, we are able to integrate macroscale anatomy with the surface features to understand the contact mechanical behaviour of adhesive projectile tongues in frogs. While the contact is quickly established by a progressive increase of the contact area from the proximal to the distal regions of the tongue, the contact release happens much slower and progresses from the outside to the inside of the tongue contact area. During detachment, we observed fibrillation—a process that is typical for PSAs [24,25]. Our data thus show that frog tongues, indeed, act as PSAs as we previously suggested [22].

**Figure 3.**
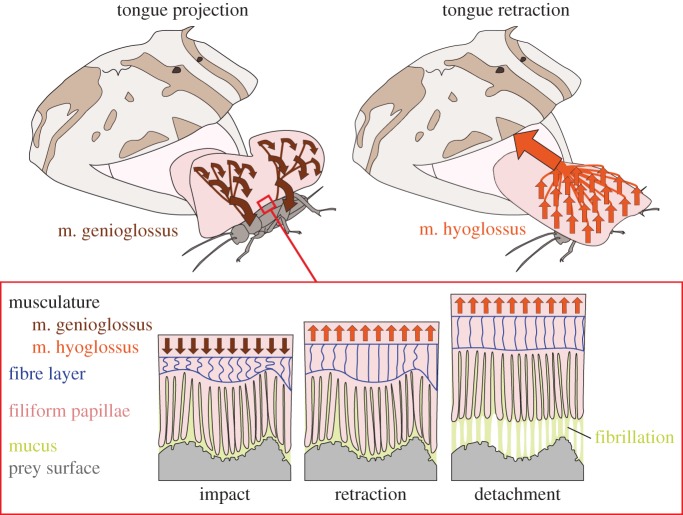
Schematic of the events on the macroscopic and microscopic level during frog tongue feeding. During projection, the tongue performs a rotational movement that is caused by the momentum of the lower jaw during depression and action of the m. genioglossus (brown arrowheads). Tongue retraction is caused by action of the m. hyoglossus (orange arrwoheads). The fibres of the m. hyoglossus are spread evenly over the tongue and insert perpendicular to the tongue surface. This arrangement of muscle fibres causes an equal force distribution over the contact area and thus allows for high pulling forces. The presence of filiform papillae and a layer of fibrous material underneath the tongue epithelium allows for a good adaptability of the tongue to variable surfaces. The entire system is submerged in mucus. If detachment occurs, fibrils of mucus still maintain contact with the prey surface and thus allow for continued pulling forces. The effect of fibrillation is predicted to benefit from the presence of filiform papillae, which will aid in anchoring the mucus fibrils.

In frogs, tongue projection is caused by a rapid opening of the lower jaw and action of the tongue-protracting muscle m. genioglossus, which will result in a rotation of the tongue around the jaw symphysis that acts as a pivotal point [19,21,29–31] (electronic supplementary material, video S3). During tongue projection, the distal parts of the tongue travel the longest distance and will hit the surface with some delay relative to the proximal regions. This time-lag between proximal and distal tongue areas explains the progression of the contact formation from proximal to distal which we observed herein (figure 3). The time to establish a full contact is approximately twice as fast as the time it takes to reach the maximum tongue impact force (39.1±19.6 ms, *n*=80), which we measured for the same frogs in a previous experiment [22]. This delay between maximum contact area and maximum impact force suggests that the tongue continues to push against the surface while it is already in full contact.

During retraction, the even distribution of m. hyoglossus muscle fibres inside the body of the tongue causes the tongue to be pulled perpendicular to the interface with the target (figure 3). Thus other than peeling, where one would expect the contact to break from one side to the other, in *Ceratophrys*, the tongue is pulled over its entire surface area simultaneously and the contact area shrinks from the outside to the inside (figure 1*h*). For peeling, the force to break a contact only depends on the width of the leading edge and the peeling angle [32]. In *Ceratophrys*, however, the tongue-pulling force depends on the size of the contact area [22]. Prevention of peeling by pulling the entire tongue perpendicular to the contact area is suggested to be a mechanism that will allow for high tongue-pulling forces before the contact breaks. During natural feeding, this will allow these frogs to lift heavy prey, such as rodents [33] or other frogs [34] off the ground.

Further, simultaneous pulling over the entire contact area in combination with detachment of the tongue from the outside to the inside of the contact area suggests that frog tongues detach by mode I, according to the classification of contact release modes by Carbone *et al*. [35]. In this mode of detachment, the flat punch contact between an adhesive material and a target breaks by crack propagation from the outside to the inside while the pulling force is evenly distributed [35]. The force needed to break a contact in this contact release mode is predicted to be independent from the presence of imperfections in the contact interface, such as dirt particles [35], which frogs will encounter during regular feeding events.

The first rapid decrease of tongue contact during retraction probably corresponds to a phase in which peak pulling forces act while the following phase of linear reduction of the contact area over time suggests constant forces. During natural feeding, the latter phase will play a minor role because for a feeding strike, the tongue will move quickly back into the mouth of the frog. Once in the mouth, other detachment mechanisms (e.g. scraping the tongue against the palate or reduction of the tongue stickiness in a moist environment) might help to release the tongue from the target. However, provoking the frogs to detach their tongues from a glass surface allows us to reveal some basic mechanisms behind the adhesiveness of the tongue in frogs. The observed pattern of a linear reduction of the contact area over time matches our previous force over time measurements in *Ceratophrys* [22] and is also known from manufactured PSAs [23,24]. In PSAs, almost constant pulling forces during detachment are caused by the process of fibrillation, i.e. the PSA is not separated instantaneously from the opposing surface but instead the contact area breaks slowly by cavities that emerge between fibrils of PSA material [23,24]. Our observation of the formation of mucus fibrils during frog tongue adhesion *in vivo* (figure 1*i* and electronic supplementary material, video S2) thus provides further evidence that frog tongues are PSAs.

However, it shall be noted that for PSAs, it was predicted that cavities first emerge from the centre of the contact area where stresses are highest [36]. In *Ceratophrys*, we observed fibrils around the periphery of the tongue contact with the glass. This pattern might be related to the flexibility of frog tongues while PSAs are usually tested on rigid probes [37,38]. This flexibility will cause the tongue to deform under tension in a way that the tongue is stretched perpendicular to the target surface, whereas its cross section and thus the contact area becomes narrower.

Fibrillation during detachment might also be related to the surface profile of the tongue in *Ceratophrys*, which is covered by numerous filiform papillae and several fungiform papillae. The later were previously described as chemoreceptors [39–42], whereas the filiform papillae are known to be the regions of mucus production in frogs [43]. Although the mechanical and chemical properties of the mucus remain to be resolved, it is likely that the interaction between the mucus and the filiform papillae is important for the adhesive mechanism of frog tongues. The presence of numerous hair-like filiform papillae on the tongue surface might increase the frictional forces acting on the leading edge of contact release during tongue retraction. A similar mechanism of higher shearing resistance by hair-like structures was previously suggested for other biological wet adhesive systems, such as the suctorial disc of the northern clingfish *Gobiesox maeandricus* [44] and octopus suction cups [45,46]. Further, comparable to a brush applying paint to a surface, the filiform papillae will interact with the mucus and fibrils are likely to form in-between neighbouring filiform papillae (figure 3). The filiform papillae are also suggested to increase the adaptability of the tongue to asperities on the opposing target surface. A high adaptability of the tongue is crucial for contact formation at any surface profile, especially given the extremely short time intervals for attachment reported here.

The biological tissue inside the tongue is supposed to contain large amounts of liquids *in vivo* (inside individual cells as well as an extracellular matrix), which will cause the tongue to have an almost constant volume under compression (i.e. during impact) and tension (i.e. during retraction). However, in the experiment described herein, the tongue appeared very flexible and deforms notably during impact and retraction, thus it might be best compared with a water-filled balloon. The presence of nanoscale-thin fibres underneath the tongue surface will further increase the adaptability of the tongue in compression by lowering its deformation resistance (figure 3). However, under tension, these fibres will add strength to the body of the tongue, which is needed to withstand the high pulling forces during retraction (figure 3).

Here, we show that the tongue of frogs is highly specialized to its extremely fast and reliable adhesive performance on multiple levels of its organization comprising the distribution of muscle fibres, the presence of microscale surface structures that interact with viscous mucus, and the internal organization of underlying material layers. Experimental data show that frog tongues act as PSAs and may provide a new model system in the development of improved biomimetic and environment-friendly adhesive materials.

## Supplementary Material

SupplementaryMaterial.pdf

## Data accessibility

Electronic supplementary material, table S1: data from the contact dynamics experiment. Electronic supplementary material, video S1: high-speed video recording of tongue impact in *Ceratophrys* sp. against a glass surface; filmed at 2000 frames per second and replayed at 12 frames per second (i.e. slowed down by a factor of approximately 167). Electronic supplementary material, video S2: high-speed video recording of tongue impact and retraction in *Ceratophrys* sp. against a glass surface; filmed at 2000 frames per second and replay speed corresponds to 90 frames per second (i.e. slowed down by a factor of 22). Electronic supplementary material, video S3: high-speed video recording of a *Ceratophrys* sp. catching a cricket; filmed at 1000 frames per second and replayed at 24 frames per second, i.e. 42 times slowed down. *Micro-CT datasets*: micro-CT datasets of the head, tongue and tongue tissue of *C. ornata* are available at the Dryad repository under the following link: (http://dx.doi.org/10.5061/dryad.066mr).
