# Supplementary material for: Frog tongue acts as muscle-powered adhesive tape
Source: R Soc Open Sci. 2015 Sep 30;2(9):150333. doi: 10.1098/rsos.150333 (PMC4593688; doi:10.1098/rsos.150333)
Supplement: SupplementaryMaterial.pdf [file rsos150333supp1.pdf]

Frog tongue acts as muscle powered adhesive tape

Thomas Kleinteich and Stanislav N. Gorb

Kiel University, Functional Morphology and Biomechanics, Am Botanischen Garten 9, 24118 Kiel, Germany

Supplementary Material

Supplementary table 1: Data from the contact dynamics experiment

Supplementary video 1: High-speed video recording of tongue impact in *Ceratophrys* sp. against a glass surface; filmed at 2,000 frames per second and replayed at 12 frames per second (i.e. slowed down by a factor of approximately 167).

Supplementary video 2: High-speed video recording of tongue impact and retraction in *Ceratophrys* sp. against a glass surface; filmed at 2,000 frames per second and replay speed corresponds to 90 frames per second (i.e. slowed down by a factor of 22).

Supplementary video 3: High-speed video recording of a *Ceratophrys* sp. catching a cricket; filmed at 1,000 frames per second and replayed at 24 frames per second, i.e. 42 times slowed down.

Supplementary table 1: Data from the contact dynamics experiment

Date	Specimen	Trial #	Duration of contact formation [ms]	Maximum contact area [mm2]	Duration of contact release [ms]	Total contact duration [ms]	% linear phase to contact release	Contact Releasing Speed [cm2 / s]	Regression statistics for linear phase of contact release						Comments
									t	DF1	DF2	Adj. r2	F	p	
**2014_09_04**	Cerato_l	1	32.5	319.14	2468	2500.5	46.60	-1.179	-474.2	1	2297	0.990	2.25E+05	2.2E-16	
**2014_09_04**	Cerato_l	3	9	436.83	1789.5	1798.5	64.26	-1.747	-594.4	1	2297	0.994	3.53E+05	2.2E-16	
**2014_09_04**	Cerato_r	1	32	169.65	416.5	448.5	48.02	-3.212	-61.06	1	397	0.904	3.73E+03	2.2E-16	
**2014_09_04**	Cerato_r	2	27	124.46	118.5	145.5	42.19	-6.232	-50.49	1	97	0.963	2.55E+03	2.2E-16	
**2014_09_16**	Cerato_r	2	13	236.53	358	371	62.85	-5.658	-81.55	1	447	0.937	6.65E+03	2.2E-16	
**2014_09_22**	Cerato_l	1	55	336.26	NA	NA	NA	-0.918	-490.5	1	3897	0.984	2.41E+05	2.2E-16	contact not fully released
**2014_09_22**	Cerato_l	2	6.5	246.68	731.5	738	34.18	-2.471	-409.1	1	497	0.997	1.67E+05	2.2E-16	
**2014_09_22**	Cerato_r	1	6	143.87	557	563	80.79	-1.661	-44.85	1	897	0.691	2.01E+03	2.2E-16	
**2014_10_14**	Cerato_l	1	66	515.52	2599.5	2665.5	61.55	-2.095	-970.9	1	3197	0.997	9.43E+05	2.2E-16	
**2014_10_14**	Cerato_r	1	17	422.23	1014.5	1031.5	49.29	-1.886	-191.2	1	997	0.973	3.66E+04	2.2E-16	
**2014_10_14**	Cerato_V	2	12.5	313.31	223	235.5	44.84	-4.140	-55.69	1	197	0.940	3.10E+03	2.2E-16	
**2014_10_20**	Cerato_r	1	9.5	436.30	926	935.5	54.00	-2.419	-292.4	1	997	0.989	8.55E+04	2.2E-16	
**2014_10_20**	Cerato_r	2	5.5	470.47	854	859.5	52.69	-3.799	-382.4	1	897	0.994	1.46E+05	2.2E-16	
**2014_10_20**	Cerato_V	1	20.5	537.65	2916	2936.5	48.01	-1.867	-580.9	1	2797	0.992	3.38E+05	2.2E-16	
**2014_10_20**	Cerato_V	2	10.5	238.31	1361	1371.5	66.13	-1.281	-314.3	1	1797	0.982	9.88E+04	2.2E-16	
**2014_10_20**	Cerato_V	3	2.5	107.37	129.5	132	38.61	-4.634	-86.88	1	97	0.987	7.55E+03	2.2E-16	
**2014_10_20**	Cerato_V	4	37.5	154.02	648	685.5	69.44	-1.390	-476.6	1	897	0.996	2.27E+05	2.2E-16	
**2014_10_28**	Cerato_r	1	22	202.27	276.5	298.5	90.42	-3.206	-88.12	1	497	0.940	7.77E+03	2.2E-16	
**2014_10_28**	Cerato_r	2	6	355.90	767	773	61.93	-2.659	-576.3	1	947	0.997	3.32E+05	2.2E-16	
**2014_10_28**	Cerato_r	3	4	265.80	452	456	60.84	-1.810	-129.2	1	547	0.968	1.67E+04	2.2E-16	
**2014_10_28**	Cerato_V	1	11	561.89	1482.5	1493.5	87.69	-2.712	-1200	1	2597	0.998	1.44E+06	2.2E-16	
**2014_10_28**	Cerato_V	2	11.5	621.44	2186.5	2198	80.04	-2.394	-877.6	1	3497	0.996	7.70E+05	2.2E-16	
**2014_11_05**	Cerato_V	2	27	402.12	2680	2707	61.57	-1.259	-472	1	3297	0.985	2.23E+05	2.2E-16	
**2014_11_05**	Cerato_V	3	17.5	178.31	430	447.5	58.14	-2.652	-70.29	1	497	0.908	4.94E+03	2.2E-16	
**2014_11_05**	Cerato_V	4	NA	498.15	2870	NA	69.69	-1.626	-1381	1	3997	0.998	1.91E+06	2.2E-16	start of contact initiation missing
